# The characteristics of postprandial glycemic response patterns to white rice and glucose in healthy adults: Identifying subgroups by clustering analysis

**DOI:** 10.3389/fnut.2022.977278

**Published:** 2022-10-31

**Authors:** An-shu Liu, Zhi-hong Fan, Xue-jiao Lu, Yi-xue Wu, Wen-qi Zhao, Xin-ling Lou, Jia-hui Hu, Xi-yi-he Peng

**Affiliations:** ^1^College of Food Science and Nutritional Engineering, China Agricultural University, Beijing, China; ^2^Key Laboratory of Precision Nutrition and Food Quality, Department of Nutrition and Health, China Agricultural University, Beijing, China

**Keywords:** clustering analysis, glycemic response pattern, white rice, glucose, glycemic index

## Abstract

**Objectives:**

Large interpersonal variability in postprandial glycemic response (PGR) to white rice has been reported, and differences in the PGR patterns during the oral glucose tolerance test (OGTT) have been documented. However, there is scant study on the PGR patterns of white rice. We examined the typical PGR patterns of white rice and glucose and the association between them.

**Materials and methods:**

We analyzed the data of 3-h PGRs to white rice (WR) and glucose (G) of 114 normoglycemic female subjects of similar age, weight status, and same ethnic group. Diverse glycemic parameters, based on the discrete blood glucose values, were calculated over 120 and 180 min. K-means clustering based on glycemic parameters calculated over 180 min was applied to identify subgroups and representative PGR patterns. Principal factor analysis based on the parameters used in the cluster analysis was applied to characterize PGR patterns. Simple correspondence analysis was performed on the clustering categories of WR and G.

**Results:**

More distinct differences were found in glycemic parameters calculated over 180 min compared with that calculated over 120 min, especially in the negative area under the curve and Nadir. We identified four distinct PGR patterns to WR (WR1, WR2, WR3, and WR4) and G (G1, G2, G3, and G4), respectively. There were significant differences among the patterns regard to postprandial hyperglycemia, hypoglycemic, and glycemic variability. The WR1 clusters had significantly lower glycemic index (59 ± 19), while no difference was found among the glycemic index based on the other three clusters. Each given G subgroup presented multiple patterns of PGR to WR, especially in the largest G subgroup (G1), and in subgroup with the greatest glycemic variability (G3).

**Conclusion:**

Multiple subgroups could be classified based on the PGR patterns to white rice and glucose even in seemingly homogeneous subjects. Extending the monitoring time to 180 min was conducive to more effective discrimination of PGR patterns. It may not be reliable to extrapolate the patterns of PGR to rice from that to glucose, suggesting a need of combining OGTT and meal tolerance test for individualized glycemic management.

## Introduction

As the risk factors of the cardiovascular disease and some cancers, diabetes, and dysglycemia are regarded as one of the most important medical concerns in most parts of the world (1). Early detection of a tendency toward diabetes, as well as the effective management of postprandial glycemic excursion, are of public health significance.

The progression from an apparent health person to a diabetic consists of multiple stages such as insulin resistance, hyperglycemia, β-cell dysfunction, β-cell mass reduction, and impaired glucose tolerance (1, 2). The oral glucose tolerance test (OGTT), which measures the postprandial glycemic response (PGR) to glucose in 120 min of an individual, is widely used to assess the insulin sensitivity, the beta-cell function, and judge an individual’s metabolic capacity to handle carbohydrate foods (3). The glycemic compromised individuals were usually classified into several category of dysglycemia and receive advice based on their OGTT results based on the fasting and glucose values within 120 min (4). However, recent studies indicated that compared with the glucose values at certain time points, the features of OGTT curve might be better associated with future end points such as diagnosed T2DM and all-cause mortality risk (5–7).

Similarly, the glycemic index (GI), which is calculated based on the incremental area under curve (iAUC) of glucose and a test food, is an extensively used parameter of the PGR curve to a certain food. However, there are evidence that the GI value and the iAUC of a food could not fully illustrate the multiple attributes of the PGR patterns among difference groups of responders (8, 9). The connotation of PGR to a food includes postprandial hyperglycemia, hypoglycemic, and glycemic variability. Compared with glucose concentration of 1 or 2 h and the iAUC, the PGR pattern, which describes the peak and the peak time, the speed of glucose dropping, the level of nadir, and the magnitude of glycemic excursion over the time, may provide more information related to pathophysiological differences of individuals (10–12). Such a set of glycemic information can be conducive not only to early identification of the individuals at risk, but also to successful management of the prediabetes and the diabetes.

White rice, a major staple food in most Asian diets and one of the important carbohydrate sources in many other regions over the world (13, 14), has caused wide concern in terms of its contribution to the overall glycemic load. However, the results of epidemiologic literature on rice consumption and risk of type 2 diabetes mellitus (T2DM) are mixed, range from positive (15), null (16, 17) to negative (18). One of the potential contributors to this inconsistency may be the large interpersonal variability in PGR to white rice, which have been investigated by previous studies in aspect of iAUC (19–21). But few of them have inspected the PGR patterns to rice. In addition, the correspondence between the individual PGR patterns to rice and glucose has not been investigated. It is yet to be confirmed that whether the postprandial glycemic curve of a glucose test can predict the PGR patterns to a rice meal.

In the present study, the data of PGRs to white rice (WR) and glucose (G) of 114 subjects in previous studies carried out in our laboratory were included for comparison with respect to their PGR characteristics. The aims of this study were: (1) to identify the subgroups based on their PGR patterns of WR and G; (2) to examine the association between the PGR patterns of WR and G; (3) to find whether the glycemic index (GI) of rice would differ among subgroups. We assumed that the PGR patterns to white rice and glucose varied even in seemingly homogenous healthy subjects and could be classified into distinguish subgroups.

## Materials and methods

### Data collection

This study was a *post-hoc* analysis. We derived the data from several acute feeding trials conducted in our laboratory in the past 5 years, and all participants signed informed consent forms for participation in these studies (22–27). The subjects of these trials were recruited through the university bulletin boards and online advertisements with similar recruitment criteria which were: (1) normal weight (BMI in the range of 18.5–25.0 kg/m^2^) healthy university students aged between 18 and 25 with normal fasting glucose and normal glucose tolerance; (2) having three meals regularly and not on diet to gain or to lose weight in the past 3 months; (3) no habit of smoking/alcohol drinking/dependency on drugs or medication; (4) not participated in competitive sports or high intensity training.

These trials used randomized, repeated measures cross-over design and same procedure. The participants consumed test meals in a randomized order. The test meals included cooked white rice (*Oryza sativa* spp. *japonica*) and glucose solution, each containing 50 g available carbohydrates. There was at least 3 days between two test sessions to ensure adequate washout. The subjects were asked not to take any test 3 days before and after the start of menstruation. One day before each trial day, the participants were instructed to refrain from excessive eating, alcohol, staying up late and strenuous exercise.

**FIGURE 1:**
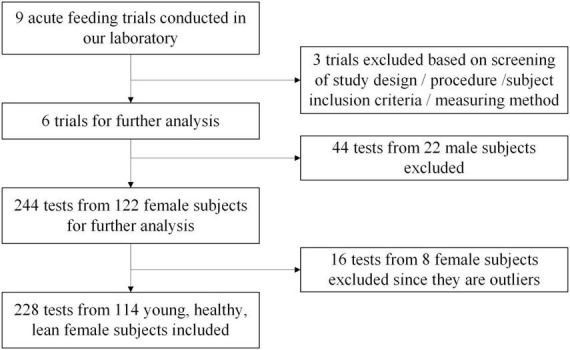
Flow diagram for data collection.

On the test day, the subjects came to the laboratory at morning after a 12 h overnight fast, and their fasting plasma glucose concentrations were tested after a short rest. Then the test meal was provided to the subjects and the food was ingested within 5–15 min. The finger prick blood samples were collected at 0 (fasting), 15, 30, 45, 60, 90, 120, 150, and 180 min. The plasma blood glucose concentrations were measured on an ONETOUCH^®^ Ultra^®^ (LifeScan Inc., Milpitas, CA, USA) glucometer using the glucose oxidase method. The fat mass was assessed by bio-impedance, using an eight-polar tactile electrode system (HBF-371, OMRON Corp., Kyoto, Japan). All the trials were approved by the Ethics Committee of China Agricultural University (ethics number 2016011, 2016012, CAUHR-2019001, CAUHR-2019002, CAUHR-2019006, CAUHR-2019007). After the selection and exclusion procedure (as shown in Figure 1), 228 tests from 114 female participants were included in the final analysis.

### Outcome

The primary measurements were glucose concentrations at 0 (fasting), 15, 30, 45, 60, 90, 120, 150, and 180 min. The PGRs were converted to the values of glucose rises from the fasting value.

The specific outcomes were the incremental areas under the curve of PGRs (iAUC), the ratio of iAUC in different periods to total iAUC (iAUC%), the maximum postprandial glucose rise (Peak), the minimum postprandial glucose rise (Nadir), the standard deviation (SD) of glycemic variability, continuous overlapping net glycemic action (CONGA1), and the negative area under the curve (NAUC). The iAUCs were calculated using the trapezoidal method, ignoring the area beneath the fasting level. The CONGA1 described the SD of the differences between any individual glucose reading and a reading recorded either 1 h previously (28). The NAUC was the area beneath the fasting level. Given that the current recommendations stipulate the blood glucose should be monitored for 2-h post-ingestion in GI determinations and OGTT, these parameters were calculated both over 120 and 180 min, respectively.

### Statistical analysis

A power calculation was conducted with the PASS 2021 (NCSS, Kaysville, UT, USA) using one-way analysis of variance allowing unequal variances, based on the mean and SD of iAUCs of four distinct glucose patterns during the OGTT observed by Hulman et al. (7). A sample size of *n* = 52 was required to provide 90% power to detect the difference in iAUC at 90% power and significance of 5% between groups. All statistical analyses were performed with SPSS version 21.0 (SPSS Inc. Chicago, IL, USA). The Identify Unusual Cases application in SPSS was used for the outlier exclusion. The parameters calculated over 180 min (iAUC_60–180_%, iAUC_180_, NAUC_180_, SD_180_, Peak_180_, Nadir_180_, and CONGA1_180_) were selected as analysis variables. The anomaly detection was performed on the datasets of white rice (WR) tests and glucose (G) tests separately. If a subject was identified as an anomalous case in any detection, the data of WR and G test would be excluded both. Comparison of glycemic parameters between test meals was done by using paired *t*-test or Wilcoxon signed-rank test.

Considering the intra-individual variability of the PGR to different test meals, cluster analysis was performed on the datasets of white rice (WR) tests and glucose (G) tests separately. After the pre-analysis, the parameters calculated over 180 min (iAUC_60–180_%, iAUC_180_, NAUC_180_, SD_180_, Peak_180_, Nadir_180_, and CONGA1_180_) were used for identification and classification of PGR patterns. We applied K-means clustering based on these glycemic parameters to identify subgroups, using squared Euclidean distance as distance measure. To ensure that the classes were in the same range, Z-score normalization was applied. To improve the reproducibility, clustering was replicated 50 times, and the outcome with lowest total sum of distances was chosen. We chose K = 4 because it led to distinct PGR patterns that represent the variation in the population. Then, differences of anthropometric characteristics were estimated by multinomial logistic regression models with class of PGR pattern as the outcome and values of BMI and fat mass as factors.

Generalized estimating equation was used to compare the difference of glucose rises at each time point between clusters. Postprandial glycemic parameters of clusters were analyzed by one-way ANOVA test or Kruskal–Wallis test. Then, we applied principal factor analysis (PFA) to summarize and visualize the responses to the parameters, and further characterize subgroups. PFA with varimax rotation was performed on the “114 × 7” matrix (114 participants × 7 parameters used in the cluster analysis) of the WR dataset and the G dataset separately. Retention of items was based on combined evaluation of the scree plot (number of factors on scree plot just before elbow) and eigenvalues over 1.0 to model factor structure (29). The factor scores were calculated by regression method. Then, the PFA was repeated on the dataset conducted by glycemic parameters calculated over 120 min (iAUC_60–120_%, iAUC_120_, NAUC_120_, SD_120_, Peak_120_, Nadir_120_, and CONGA1_120_). One-way ANOVA test was applied to compare the factor scores between subgroups. *P*-values of < 0.05 were considered statistically significant. Furthermore, simple correspondence analysis (SCA) was performed on clustering categories of WR and G.

## Results

Baseline characteristics of participants are displayed in Table 1. The anthropometric measurements of the participants were within the acceptable normal limits for BMI, fasting blood glucose.

**TABLE 1:** Baseline characteristics of participants (*n* = 114).

	Mean	SD
Age, years	22	2
BMI, kg/m^2^	20.8	2.0
Fat mass, %	25.2	4.5
Height, cm	164.7	7.1
Weight, kg	56.4	8.5
Fasting glucose, mmol/L	5.1	0.4

### Inter-individual variation in postprandial responses

**FIGURE 2:**
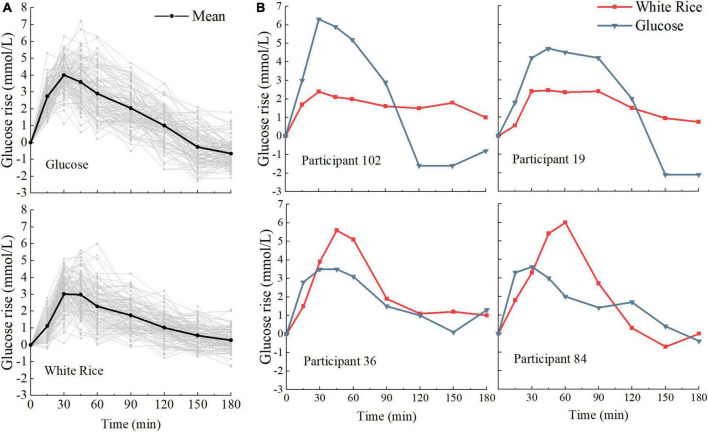
Variation in postprandial responses. **(A)** Inter-individual variation in glucose postprandial responses to white rice (WR) and glucose (G) (*n* = 114). **(B)** Example of the postprandial glycemic response (PGR) to meals for four participants exhibiting opposite PGR patterns.

**FIGURE 3:**
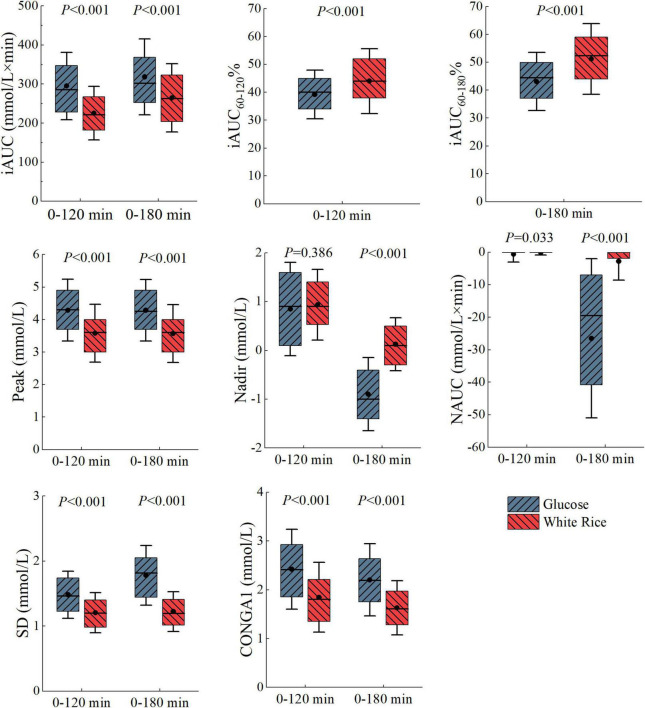
Boxplot of glycemic parameters calculated over 180 and 120 min. The dots indicate the mean, the line indicate the media, **top** of the box indicate the 75th percentile, **bottom** of the box indicate the 25th percentile and the error bars indicate the mean ± SD. Statistical significance between white rice (WR) test and glucose (G) test is marked.

We examined interpersonal variability in the PGRs to WR and G. When comparing the PGRs of each person to the same meal, we found high interpersonal variability across all postprandial time points (Figure 2A). There was also a broad range of individual response to specific outcomes (Figure 3), especially in iAUC_180_ (CV = 32.97 for WR, 30.50 for G), CONGA1_180_ (CV = 34.07 for WR, 33.61 for G), CONGA1_120_ (CV = 38.65 for WR, 33.91 for G), and NAUC_180_ (interquartile range for WR, 33.76 for G). There was difference between the GI_180_ of white rice based on iAUC_180_ (86 ± 28) and the GI_120_ based on iAUC_120_ (80 ± 25). The large interpersonal differences in PGRs are also evident in that the type of meal that induced the highest PGR differs across participants and that different participants might have opposite PGRs to the pair of meals (Figure 2B).

### Clustering subgroups

When comparing the glycemic parameters of WR and G test, the difference was more distinct in outcomes calculated over 180 min (Figure 3). Hence, the cluster analysis was carried out on parameters over 180 min, which were iAUC_60–180_%, iAUC_180_, NAUC_180_, SD_180_, Peak_180_, Nadir_180_, and CONGA1_180_.

#### Glucose subgroups

**FIGURE 4:**
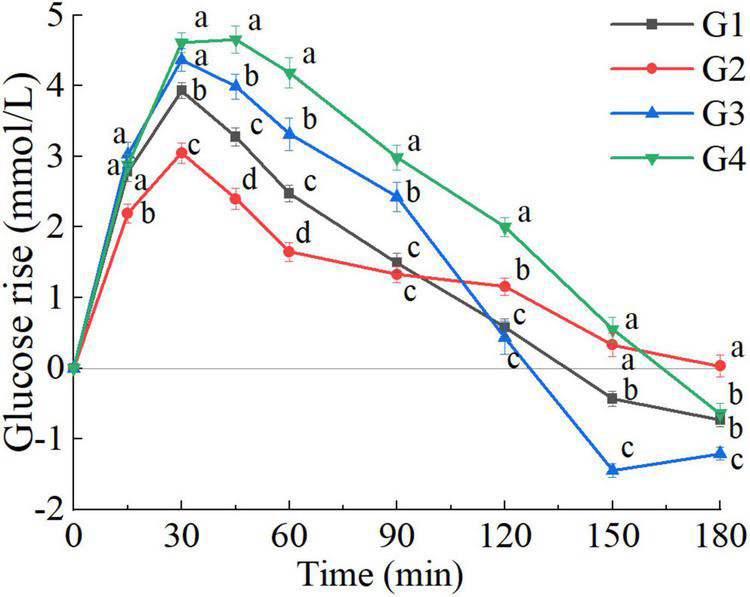
Postprandial glycemic response (PGR) to glucose of G clusters (G1, G2, G3, G4). a, b, c, d, used for comparison between subgroups at that time point (*P* < 0.05). The error bars indicate the mean ± SE.

The clustering carried out on PGRs to glucose divided the participants into four subgroups (G1, G2, G3, G4), and the silhouette coefficient was 0.241. As shown in Figure 4, striking differences appeared in all postprandial time points (*P* < 0.05). Immediately after the meal, the rate of blood glucose rise was found to be higher in G1, G3, and G4 than G2, particularly in G4. According to the average PGR curves, the time to peak of G1, G2, and G3 was 30 min, while G4 was 45 min. In the wake of a rapid drop of blood glucose level, evident hypoglycemic troughs appeared in G1 and G3 (0 vs. 150, 180 min, *P* < 0.005). Postprandial blood glucose values of G2 were above the fasting level throughout the test session (0 vs. 180 min, *P* = 0.843). In G4, only the glucose value at 180 min was lower than the fasting value. No significant difference was found in terms of BMI and fat mass distribution among the four subgroups.

##### Postprandial glycemic response pattern characteristics of glucose clusters

###### Principal factor analysis of glycemic parameters calculated over 120 min.

**FIGURE 5:**
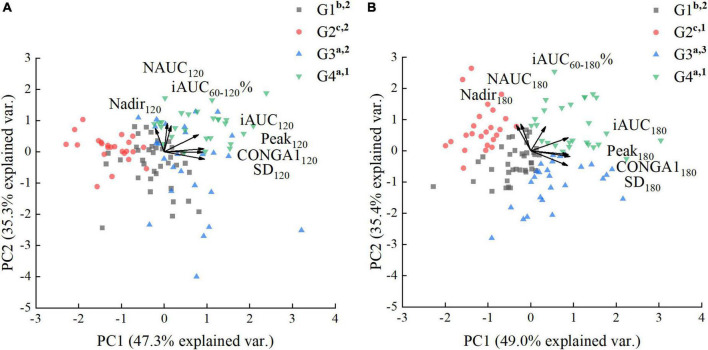
Principal factor analysis loading and score plots of glucose (G) clusters. **(A)** Principal factor analysis (PFA) based on parameters calculated over 120 min. **(B)** PFA based on parameters calculated over 180 min. The loadings of the parameters are shown with arrows, and the scores of the participants are shown as dots in the background. a, b, c used for comparison of PC1 scores (*P* < 0.05). 1–4 used for comparison of PC2 scores (*P* < 0.05).

There were differences in all glycemic parameters among the clusters (Table 2). However, univariate analysis was inappropriate because of the correlation between the glycemic parameters. Therefore, PFA was applied to summarize and visualize the responses to all the observed parameters, and further characterize subgroups. This multivariate visualization is complementary to the PGR curve. The examination of the scree plot and eigenvalues suggested that two factors best fit the data. The factors extracted (PC1, PC2) accounted for 82.6% of the variance (Figure 5A). The loadings of iAUC_120_, SD_120_, Peak_120_, and CONGA1_120_ in PC1 were greater than PC2, suggesting that PC1 score was related to the amplitude of PGR and the glycemic variability. As shown in Figure 5A, the PC1 score was positively correlated with iAUC_120_, SD_120_, Peak_120_, and CONGA1_120_. The loadings of iAUC_60–120_%, NAUC_120_, and Nadir_120_ in PC2 were greater than PC1, suggesting that PC2 score was related to the rate of glucose decline and the hypoglycemic excursion, while the PC2 score was positively associated with iAUC_60–120_%, NAUC_120_, and Nadir_120_. The G4 and G3 clusters possessed the high amplitude of PGR to G (highest PC1 score). In contrast, G2 cluster was able to sustain a small elevation with lowest PC1 score. However, when comparing the PC2 scores related to the rate of glucose decline and the hypoglycemic fluctuation, no significant difference was found between G1, G2 and G3 clusters (*P* > 0.05).

**TABLE 2:** Body mass index (BMI), fat mass, and glycemic parameters calculated over 120 min of glucose (G) clusters (*n* = 114).

	G1 (*n* = 37)	G2 (*n* = 24)	G3 (*n* = 26)	G4 (*n* = 27)
BMI	20.4 (1.8)	20.8 (1.7)	21.3 (2.3)	20.7 (2.1)
Difference^1^	0.99 (0.72–1.36)	1.12 (0.85–1.67)	1.33 (0.96–1.84)	Reference
Fat mass	24.9 (3.9)	24.7 (4.7)	25.3 (4.3)	25.9 (4.9)
Difference^1^	0.95 (0.82–1.10)	0.90 (0.77–1.05)	0.92 (0.78–1.05)	Reference
CONGA1_120_	2.3 (0.5)^b^	1.5 (0.5)^c^	2.8 (0.8)^a^	3.0 (0.7)^a^
iAUC_60–120_%	34.5 (8.5)^b^	39.1 (6.3)^b^	38.9 (10.0)^b^	45.9 (4.8)^a^
iAUC_120_	258.5 (44.9)^c^	208.0 (36.6)^d^	323.9 (68.7)^b^	394.3 (61.1)^a^
NAUC_120_	0.0 (0.1)^ab^	0 (0.0)^b^	0.0 (1.4)^a^	0.0 (0.0)^b^
Peak_120_	4.1 (0.6)^b^	3.2 (0.5)^c^	4.7 (0.7)^a^	5.1 (0.8)^a^
Nadir_120_	0.5 (0.7)^b^	0.8 (0.4)^b^	0.4 (1.2)^b^	1.8 (0.7)^a^
SD_120_	1.5 (0.2)^b^	1.0 (0.2)^c^	1.7 (0.3)^a^	1.7 (0.3)^a^

Values are mean (SD), except that NAUC_120_ is median (first quartile, third quartile).

^1^Difference (95% CI) from multinomial logistic regression models. a, b, c, d, used for comparison of glycemic parameters between groups based on one-way ANOVA test or Kruskal–Wallis test (*P* < 0.05).

###### Principal factor analysis of glycemic parameters calculated over 180 min.

There was more distinct difference in parameters calculated over 180 min, especially in the NAUC and SD (Table 3). For comparing the parameters calculated over 180 min between G clusters, the PFA was carried on the dataset conducted by iAUC_60–180_%, iAUC_180_, NAUC_180_, SD_180_, Peak_180_, Nadir_180_, and CONGA1_180_. The factors extracted (PC1, PC2) accounted for 84.4% of the variance (Figure 5B). The PC1 score was positively correlated with iAUC_180_, SD_180_, Peak_180_, and CONGA1_180_, while the PC2 score was positively associated with iAUC_60–180_%, NAUC_180_ and Nadir_180_. Unlike the PFA based on glycemic parameters calculated over 120 min, G clusters can be separated into four parts in the score plot clearly according to the PFA based on glycemic parameters calculated over 180 min (Figure 5B). Compared with F-Statistic of ANOVA performed on PC2 scores derived from the parameters calculated over 120 min (12.7), greater F-Statistic (46.9) indicated larger between-group variance. The highest glycemic variability observed in G3 clusters was reflected by the highest PC2 score and lowest PC1 score. The G4 cluster possessed not only the high amplitude of PGR to G (highest PC1 score), but also the low rate of blood glucose dropping (highest PC2 score). On the contrary, G2 clusters were capable of sustaining a mild increase and a slow post-peak decline of blood glucose, reflected by the lowest PC1 score and greater PC2 score. The glucose caused a moderately high rise of blood glucose but a wild hypoglycemic fluctuation in G1 clusters indicated by lower PC1 score and PC2 score.

**TABLE 3:** Glycemic parameters calculated over 180 min of four glucose (G) clusters (*n* = 114).

	G1	G2	G3	G4
CONGA1_180_	2.0 (0.4)^b^	1.3 (0.4)^c^	2.7 (0.6)^a^	2.8 (0.5)^a^
iAUC_60–180_%	36.6 (9.1)^c^	47.0 (8.4)^ab^	39.8 (10.5)^bc^	51.6 (5.5)^a^
iAUC_180_	268.8 (49.4)^c^	242.5 (51.9)^c^	330.0 (72.5)^b^	442.9 (71.7)^a^
NAUC_180_	–25.5 (23.4)^b^	–2.2 (10.7)^a^	–57.8 (32.0)^c^	–8.0 (18.3)^a^
Peak_180_	4.1 (0.6)^b^	3.2 (0.5)^c^	4.7 (0.7)^a^	5.1 (0.8)^a^
Nadir_180_	–1.0 (0.4)^a^	–0.2 (0.7)^a^	–1.6 (0.4)^b^	–0.7 (0.8)^a^
SD_180_	1.7 (0.2)^c^	1.2 (0.2)^d^	2.2 (0.3)^a^	2.0 (0.4)^b^

Values are mean (SD), except that NAUC_120_ is median (first quartile, third quartile). a, b, c, d, used for comparison between groups (*P* < 0.05).

#### White rice subgroups

**FIGURE 6:**
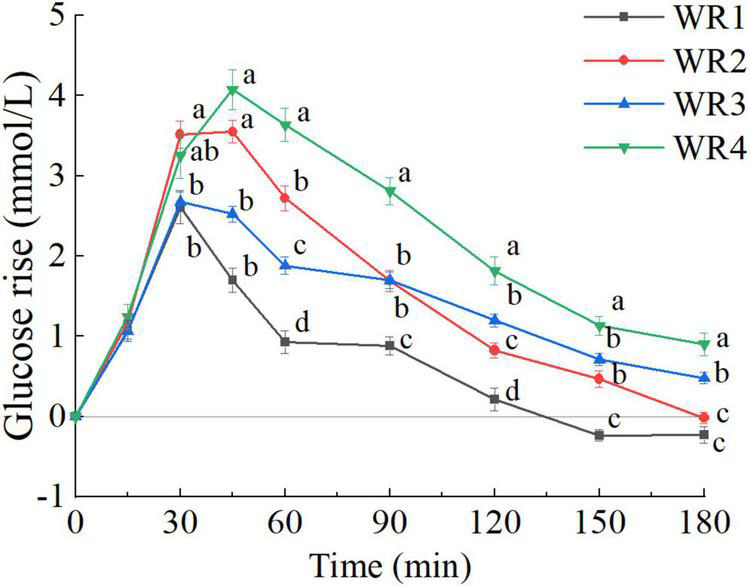
Postprandial glycemic response (PGR) to white rice of white rice (WR) clusters (WR1, WR2, WR3, WR4). a, b, c, d, used for comparison between subgroups at that time point (*P* < 0.05). The error bars indicate the mean ± SE.

The cluster analysis identified four subgroups based on PGRs to white rice (WR1, WR2, WR3, WR4), and the silhouette coefficient was 0.277. As shown in Figure 6, there were significant differences across all postprandial time points except at 15 min (*P* < 0.05). The white rice caused steeper glucose rise in WR2 and WR4 than WR1 and WR3 in the first 30 min, while the subsequent fall in glucose after 45 min was more rapid in WR1 and WR2 than WR3 and WR4. According to the average PGR curves, the time to peak was 30 min except for WR4, which was 45 min. Compared with the fasting values, both the WR3 and WR4 ended with a higher glucose concentration (0 vs. 180 min, *P* < 0.01), while the WR2 reverted to the fasting level at 180 min (0 vs. 180 min, *P* = 0.792). However, the WR1 cluster showed prolonged negative incremental glucose value after 120 min (0 vs. 150, 180 min, *P* < 0.05). No significant difference was found in terms of BMI and fat mass distribution among the four subgroups.

##### Postprandial glycemic response pattern characteristics of white rice clusters

###### Principal factor analysis of glycemic parameters calculated over 120 min.

**FIGURE 7:**
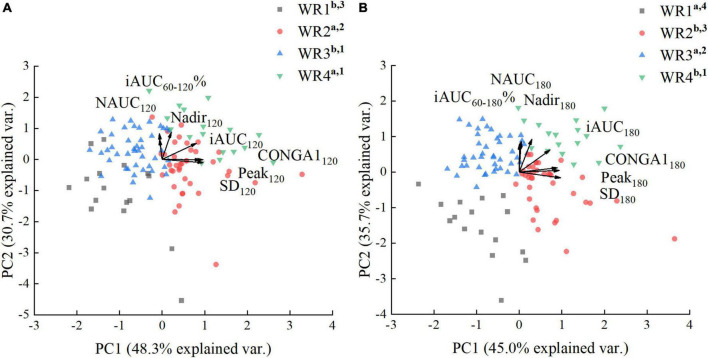
Principal factor analysis loading and score plots of white rice (WR) clusters. **(A)** Principal factor analysis (PFA) based on parameters calculated over 120 min. **(B)** PFA based on parameters calculated over 180 min. The loadings of the parameters are shown with arrows, and the scores of the participants are shown as dots in the background. a, b, c used for comparison of PC1 scores (*P* < 0.05). 1–4, used for comparison of PC2 scores (*P* < 0.05).

There were differences in all glycemic parameters among the clusters (Table 4). The factors extracted (PC1, PC2) accounted for 79.1% of the variance (Figure 7A). The PC1 score was positively correlated with iAUC_120_, SD_120_, PEAK_120_, and CONGA1_120_, while the PC2 score was positively associated with iAUC_60–120_%, NAUC_120_, and LOW_120_. WR2 clusters displayed the most oscillating glucose levels, characterized by high PC1 score and relatively low PC2 score. WR4 clusters showed constant hyperglycemia with higher PC1 and PC2 score. However, when comparing the rate of glucose decline and the hypoglycemic fluctuation (PC2 scores), no significant difference was found between WR3 and WR4 clusters (*P* > 0.05). What’s more, there was no significant difference in GI_120_ between WR2, WR3, and WR4 (Table 4).

**TABLE 4:** Body mass index (BMI), fat mass, and glycemic parameters calculated over 120 min of white rice (WR) clusters (*n* = 114).

	WR1 (*n* = 16)	WR2 (*n* = 38)	WR3 (*n* = 43)	WR4 (*n* = 17)
BMI	20.4 (1.4)	20.6 (1.9)	21.1 (2.3)	20.4 (1.72)
Difference^1^	1.09 (0.70–1.69)	1.08 (0.75–1.56)	1.31 (0.92–0.87)	Reference
Fat mass	24.3 (4.1)	25.4 (4.4)	25.3 (4.5)	25.27 (4.5)
Difference^1^	0.93 (0.77–1.12)	0.99 (0.84–1.16)	0.94 (0.80–1.1)	Reference
CONGA1_120_	1.1 (0.5)%^b^	2.3 (0.6)%^a^	1.5 (0.3)%^b^	2.4 (0.7)%^a^
iAUC_60–120_%	34.9 (12.6)%^c^	47.4 (10.5)%^b^	56.9 (8.3)%^a^	60.6 (8.41)%^a^
iAUC_120_	130.5 (30.6)%^d^	247.3 (46.8)%^b^	203.6 (40.8)%^c^	321.6 (39.4)%^a^
NAUC_120_	0.0 (0.6)%^b^	0.0 (0.0)%^a^	0.0 (0.0)%^a^	0.0 (0.0)%^a^
Peak_120_	2.7 (0.7)%^b^	4.1 (0.6)%^a^	3.0 (0.6)%^b^	4.5 (0.7)%^a^
Nadir_120_	0.2 (0.5)%^c^	0.8 (0.6)%^b^	1.0 (0.6)%^b^	1.8 (0.7)%^a^
SD_120_	0.9 (0.2)%^b^	1.4 (0.2)%^a^	1.0 (0.2)%^b^	1.5 (0.2)%^a^
GI_120_*	59 (19)%^b^	86 (27)%^a^	77 (19)^ab^	93 (24)%^a^

Values are mean (SD), except that NAUC_120_ is median (interquartile range).

^1^Difference (95% CI) from multinomial logistic regression models. a, b, c, d, used for comparison between groups based on one-way ANOVA test or Kruskal–Wallis test (*P* < 0.05).

*The GI of white rice based on iAUC_120_.

###### Principal factor analysis of glycemic parameters calculated over 180 min.

The factors extracted (PC1, PC2) accounted for 80.7% of the variance (Figure 7B). The PC1 score was positively correlated with iAUC_180_, SD_180_, Peak_180_, and CONGA1_180_, while the PC2 score was positively associated with iAUC_60–180_%, NAUC_180_, and Nadir_180_. As shown in Figure 7B, WR clusters can be separated into four parts in the score plot distinctly. Moreover, the F-Statistic of ANOVA performed on PC2 scores was 63.5, which was greater than the F-Statistic of ANOVA performed on PC2 scores derived from the parameters calculated over 120 min (19.3), indicating larger between-group variance. The WR1 clusters experienced more hypoglycemia with the lowest PC2 score and greatest NAUC_180_, while the GI_180_ based on WR1 clusters was the lowest. WR3 clusters had lower PC1 score and less postprandial glucose dips (higher PC2 score), indicating a stable PGR to WR. Compared with the GI_180_ calculated based on the WR1 and WR3 clusters, the mean value and the SD of GI_180_ derived from the WR2 and WR4 were significantly higher (Table 5).

**TABLE 5:** Glycemic parameters calculated over 180 min of four white rice (WR) clusters (*n* = 114).

	WR1	WR2	WR3	WR4
CONGA1_180_	1.1 (0.4)%^b^	1.9 (0.4)%^a^	1.3 (0.3)%^b^	2.2 (0.4)%^a^
iAUC_60–180_%	34.9 (12.6)%^c^	47.4 (10.5)%^b^	56.9 (8.3)%^a^	60.6 (8.41)%^a^
iAUC_180_	135.3 (33.7)%^c^	276.4 (51.0)%^b^	250.3 (56.2)%^b^	396.2 (49.0)%^a^
NAUC_180_	–12.2 (10.2)%^c^	–0.9 (3.4)%^b^	0.0 (0.0)%^a^	0.0 (0.0)%^a^
Peak_180_	2.7 (0.7)%^b^	4.1 (0.6)%^a^	3.0 (0.6)%^b^	4.5 (0.7)%^a^
Nadir_180_	–0.4 (0.3)%^c^	0.2 (0.4)%^c^	0.3 (0.4)%^b^	0.8 (0.4)%^a^
SD_180_	1.0 (0.2)%^b^	1.5 (0.2)%^a^	1.0 (0.1)%^b^	1.5 (0.2)%^a^
GI_180_*	57 (17)%^c^	90 (30)^ab^	86 (20)%^b^	107 (29)%^a^

Values are mean (SD), except that NAUC_180_ is median (interquartile range). a, b, c used for comparison between groups (*P* < 0.05).

*The GI of white rice based on iAUC_180_.

### Simple correspondence analysis performed on clustering categories of white rice and glucose

**FIGURE 8:**
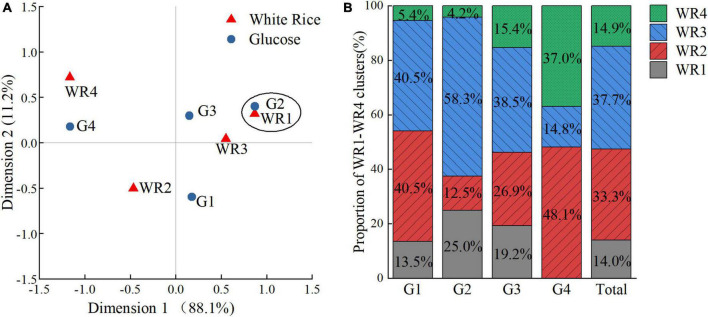
The relationships between clustering categories of white rice (WR)and glucose (G). **(A)** Individual correspondence maps between clustering categories of WR and G. **(B)** The proportions of WR1–WR4 clusters in G1–G4 clusters and the entire subjects.

The results of the Pearson’s chi-square test and Monte Carlo’s exact test revealed the significant dependence between the clustering categories of WR and G (*P* < 0.001, Cramer’s V = 0.304). Thus, SCA is applied to determine the relationships between categories of specified variables. The most important output of the analysis is a correspondence map (Figure 8A), which clearly shows the categories of analyzed variables, their mutual similarity and differences, or associations with categories of other variables. The closer two points located in the correspondence map, and the farther away the points deviated from the origin, the stronger the mutual dependence of the categories were.

As shown in Figure 8A, significant dependence was found between the clustering categories of WR1 and G2 sustaining a mild rise of blood glucose after the ingestion. Moderate dependence was found between the clustering categories of WR3 and G2, both characterized by less hyperglycemic and hypoglycemic fluctuation. In contrast, the percentage of WR2 clusters or WR4 clusters to G4 group was much larger than average either (Figure 8B), while WR2 and WR4 clusters were comparable in terms of large amplitudes of PGR but significantly different in terms of postprandial glucose dips.

As shown in Figure 8B, the share of WR2 and WR3 clusters took dominance in the G1 group, while the WR2 and WR3 clusters possessed the opposite PGR patterns to WR. In G3 group, which had the greatest glycemic excursion, the shares of the four WR subgroups were relatively balanced, while the proportion of WR1 or WR3 clusters was measurably larger than average.

## Discussion

The data of the current study showed that PGRs to the same food were highly variable across individuals even in healthy subjects of the same gender and ethnic group, similar age. Cluster analysis revealed that the subjects could be classified into distinct subgroups according to their PGR patterns to glucose and white rice. The results indicated that extending the glucose tolerance test to 180 min would be helpful to detect the possible hypoglycemic troughs after 120 min characterized in some subgroups.

In order to capture the information inherent in the PGR pattern, we used diverse parameters derived from the PGR curve. Not only those represented the elevation of blood glucose (Peak and iAUC), but also those related to the rate of post-peak decline (iAUC_60–120_% and iAUC_60–180_%), the hypoglycemia (NAUC and Nadir) and the glycemic variability (SD and CONGA) were included.

The study demonstrated that the rate of glucose decline and the NAUC could be regarded as an important aspect when differentiating the responders’ PGR patterns. The hypoglycemic variability, characterized by the rapid decline in blood glucose and a nadir below the baseline, was one of the deleterious effects of vacillating glucose levels. In the diabetic, frequent severe hypoglycemia was associated with increased risks of cardiovascular events (30), brain damage (31), retinopathy (32), and death (33). In non-diabetic persons, severe hypoglycemia after OGTT indicates the susceptibility to metabolic disorders (34). Postprandial glycemic dip in healthy subjects is also found to be able to predict appetite and energy intake (35). What’ more, there is evidence that the hyperglycemia after recovery from hypoglycemia leads to worsened endothelial function, increased oxidative stress and inflammation in both healthy individuals and the diabetic (36). Given that the most hypoglycemic values appeared after 120 min, the classification of blood glucose patterns based on 180 min blood glucose data is relevant for the management of individual PGR and contribute to the precision nutrition.

Oral glucose tolerance test is used to detect the status of glucose tolerance based on the fasting plasma glucose and 2 or 3-h plasma glucose after meal. Previous study suggested that the analysis of PGR patterns during OGTT might help to extract metabolic information and identify risks of diseases. Using the latent class trajectory analysis, Hulman et al. identified different glucose response patterns based on the shape of blood glucose curves and found that it was the PGR patterns, rather than the fasting plasma glucose and 2-h plasma glucose, that were remarkably associated with the risk of future diabetes and all-cause mortality (7, 12, 37). Froslie et al. (11) applied a functional principal component analysis to OGTT data and identified the typical temporal morphology that associated with high risk of gestational diabetes later in pregnancy. Similar to the previous studies, by cluster analysis and PFA, we observed four representative PGR patterns to glucose, i.e., the monophasic type (G1), the smooth type (G2), the biphasic oscillating type (G3), and the hyperglycemia type (G4).

In normoglycemic insulin sensitive individuals, after digestion of a bolus dosage of glucose, the blood glucose concentration increase initiates the pancreatic β-cells to secrete insulin with a biphasic pattern, results in a quick first-phase peak and a slowly rising second phase (38). The rise in blood glucose and insulin concentrations suppress the endogenous glucose production promptly, and then stimulate the uptake of glucose from peripheral tissues gradually (39). If the blood glucose level is too low, the glucagon secretion will induce the endogenous glucose production to restore the glucose homeostasis.

The equilibrium of the glucose-insulin (G-I) dynamic is reflected by an early and low peak (12), a steep slope of the decrease (6), and the nadir around the fasting level of PGR curve (40), while the G-I-related dysregulations will affect the PGR patterns in turn. We would speculate that the late and high glucose peak, a slow post-peak decline of blood glucose in G4 indicated the risk of hepatic and peripheral insulin resistance (41, 42), a weak first phase insulin secretion (43), and the lack of compensatory second phase insulin secretion (44). The coexisted high peak, rapid post-peak decline, and hypoglycemic trough in G3 implied an exaggerated second phase insulin secretion (45, 46) compensating for the inadequate first insulin secretion (47, 48) and impaired hepatic insulin sensitivity (39).

A number of studies demonstrated that genetic risk (49, 50), demographic and lifestyle factors (12) contribute to the variation of PGR pattern as deeper reasons. In addition, the rate of gastric emptying (51) and glucose absorption (52) as well as the release of incretin hormones (41, 53) may influence G-I control system and further affect the shape of the PGR curve. Hence, better understanding of glycemic patterns to glucose might allow more comprehensive assessment of the metabolic status and help to identify high-risk individuals by a simple OGTT test.

In line with the previous studies that examined individual PGRs (54, 55), we found great individual variability in PGR to glucose and rice even among the relatively homogenous subjects with similar baseline glucose value. Some research suggested that a person’s glycemic response is the result of glucose scaling to the individual (54). However, we found that the PGR pattern of glucose did not correspond to the PGR pattern of white rice exactly. The white rice elicited four distinct PGR patterns, which were the hypoglycemia type (WR1), the smooth type (WR3), the oscillating type (WR2), and the hyperglycemia type (WR4). For those who showed prolonged hyperglycemia in the glucose test (G4), merely 14.8% of them in fact had a stable glycemic response to white rice (WR3). Among those who had the best glycemic stability in glucose test (G2), only 16.7% of them were characterized by hyperglycemia after rice ingestion (WR2 and WR4). However, in the largest G subgroup (G1), and the cluster with the greatest glycemic variability (G3), the heterogeneous PGR patterns to rice made it almost impossible to predict a subject’s real PGR pattern to rice meal by OGTT.

Considering the good homogeneity of subjects, we postulate that the discrepancy between the individual PGR patterns of G and WR can be explained by the disparate digestive process of rice and glucose. Factors including salivary α-amylase activity (56), chewing patterns (57, 58), gastric emptying (51), pancreatic α-amylase activity and the impact of the food texture properties (59), and non-carbohydrate nutrients (60) might make differences to the bioavailability of carbohydrate food, the rate of gastrointestinal glucose diffusion and absorption, as well as the secretion of incretin hormones and insulin (61, 62).

In the present study, we found that a moderate proportion of the subjects, who showed great glycemic variability during G test, achieved relatively mild PGR in WR test. If they consume mixed meals consist of rice, green vegetable and protein food, they will be likely to be able to keep the PGR and the HbA1c at bay (63). Hence, in regions taking white rice as the major carbohydrate food, a combination of the OGTT, HbA1c and a white rice tolerance test may be instrumental for individualized glycemic management (64). What’s more, growing body of research have shown that the meal tolerance test could provide reliable estimation of beta-cell function and insulin resistance (65–68). Compared with other carbohydrate reference food for the meal tolerance test, the white rice meal has the advantages of good availability, high acceptability, and easy standardization.

In our study, the average GI_120_ of white rice (*O. sativa* spp. *japonica*) was 80 ± 25, close to the values reported by Atkinson et al. (69) and Yang et al. (70). However, the WR1 clusters marked difference in obviously lower GI_120_. Given the high interpersonal variability in PGR, generally grading of the japonica type white rice as “high GI food” based on the average GI may not apply to a part of the individuals. No difference was found between the GI_120_ based on WR2 and WR3, in spite of the fact that the two clusters had different PGR patterns. It is notable that the GI calculated based on the 180 min data achieved significant differences among the WR1, WR3, and WR4 subgroups, while the GI based on the 120 min data failed to differentiate the WR3 from WR4 subgroups.

Though the GI was widely used as an indicator of the quality of carbohydrate foods, the certainty of evidence for the relationship between GI and clinical outcomes was graded as low (71). This contradictory might be explained by the fact that the GI is calculated only by the iAUC in 120 min, which might not be enough to represent the PGR patterns in 180 min and beyond, which were affected by both the characteristics of the foods and the type of the subjects. Previous studies observed that the shapes of the PGR curve of foods with comparable GI could vary considerably, especially when it comes to glycemic troughs (72). Recently, based on continuous blood glucose monitoring data, new indicators such as glycemic deviation index (GDI) was developed to integrate the characteristics of the glycemic numerical value and variability, and the possibility of severe hyperglycemia/hypoglycemia (73). As most hypoglycemic episode occurred after 120 min, it is expected that some new index of glycemic stability, which includes negative area under the glycemic curve after 120 min and beyond to fully describe the glycemic variability elicited by food items.

No significant relationship between anthropometric characteristics and PGR pattern was found in this study. Since the subjects consisted of pure young, lean and healthy female subjects, living in the same environment, this study had a relatively small inter-individual variability of BMI (CV = 9.54) and fat mass (CV = 17.87). The uncollected data such as body visceral fat and lean body mass, physical activity level, lifestyle factors, genetic backgrounds, gut microbiome, which varied even in the relatively homogeneous subjects, might affect PGR pattern and need to be explored in future study.

To our knowledge, the current study is the first to classify the PGR patterns of white rice in healthy people, which might be helpful to give insight into the individual PGR to white rice. We used multiple parameters derived from the glucose curves and compared the results based on both 120 and 180 min including those associated with glycemic dips to render a full picture of the PGR patterns. Since the subjects in this study are of same ethnic group and gender group, lived in the dormitories of the same campus, and dined in the same several dining halls, the lifestyle confounders and inter-person variability were minimized. However, even if the trials included are well consistent in terms of study procedure and setting, and the management of subjects, the inter-day glycemic variation might still exist. It is suggested to take repeated measures of individual PGR to the same food. The results may not be applied to people in the diabetic, male subjects and other ethnic groups. The number of the subjects in this study is still limited. Besides, the rice sample used in the study was the japonica type rice prepared by rice cooker, which prevailed in the northeast Asia. The PGR pattern to white rice of indica type or prepared with other procedures is yet to be explored. The insulin and incretin responses of different PGR patterns, which are crucial for understanding the underlying mechanism, were not included in this analysis.

In conclusion, the present study identified four typical PGR patterns to glucose and four typical PGR patterns to white rice by cluster analysis and PFA, indicating high interpersonal variability in PGR pattern to a certain kind of food. Each given subgroup of PGR to G presented multiple patterns of PGR to WR, suggesting a need of combining the glucose tolerance test and white rice tolerance test in rice culture regions. Compared with the parameters calculated based on the postprandial 120 min curve, those based on 180 min curve might be more effective for discriminating the PGR patterns, as it better characterized the hypoglycemic part of the curve. Since it is not accurate to extrapolate the PGR patterns to a certain food only from an OGTT in many subjects, further studies are expected to understand the glycemic variability elicited by major carbohydrate food items for effective daily glycemic management.

## Data availability statement

The raw data supporting the conclusions of this article will be made available by the authors, without undue reservation.

## Ethics statement

The studies involving human participants were reviewed and approved by China Agricultural University Ethics Committee. The patients/participants provided their written informed consent to participate in this study.

## Author contributions

A-SL collected and analyzed the data and drafted the manuscript, tables, and figures. Z-HF proposed the research idea and revised the manuscript. X-JL, W-QZ, Y-XW, X-LL, J-HH, and X-Y-HP helped to collect the data. All authors contributed to the article and approved the submitted version.

## Abbreviations

PGR: postprandial glycemic response
OGTT: oral glucose tolerance test
WR: white rice
G: glucose
iAUC: incremental area under the curve
GI: glycemic index
BMI: body mass index
SD: standard deviation
CONGA: continuous overlapping net glycemic action
NAUC: negative area under the curve
PFA: principal factor analysis
SCA: simple correspondence analysis
G-I: glucose-insulin.
